# A Novel Butanol Tolerance-Promoting Function of the Transcription Factor Rob in *Escherichia coli*

**DOI:** 10.3389/fbioe.2020.524198

**Published:** 2020-09-22

**Authors:** Zhiquan Wang, Tingli Xue, Dongsheng Hu, Yuanyuan Ma

**Affiliations:** ^1^Biomass Conversion Laboratory, R&D Center for Petrochemical Technology, Tianjin University, Tianjin, China; ^2^Department of Biochemical Engineering, School of Chemical Engineering and Technology, Tianjin University, Tianjin, China; ^3^Collaborative Innovation Centre of Chemical Science and Engineering, and Key Laboratory for Green Chemical Technology, Tianjin University, Tianjin, China; ^4^State Key Laboratory of Biobased Material and Green Papermaking, Qilu University of Technology, Shandong Academy of Sciences, Jinan, China; ^5^Frontier Technology Institute, Tianjin University, Tianjin, China

**Keywords:** butanol, tolerance, *rob*, RNA-Seq, function investigation, acetate

## Abstract

Producing high concentrations of biobutanol is challenging, primarily because of the toxicity of butanol toward cells. In our previous study, several butanol tolerance-promoting genes were identified from butanol-tolerant *Escherichia coli* mutants and inactivation of the transcriptional regulator factor Rob was shown to improve butanol tolerance. Here, the butanol tolerance characteristics and mechanism regulated by inactivated Rob are investigated. Comparative transcriptome analysis of strain DT*rob*, with a truncated *rob* in the genome, and the control BW25113 revealed 285 differentially expressed genes (DEGs) to be associated with butanol tolerance and categorized as having transport, localization, and oxidoreductase activities. Expression of 25 DEGs representing different functional categories was analyzed by quantitative reverse transcription PCR (qRT-PCR) to assess the reliability of the RNA-Seq data, and 92% of the genes showed the same expression trend. Based on functional complementation experiments of key DEGs, deletions of *glgS* and *yibT* increased the butanol tolerance of *E. coli*, whereas overexpression of *fadB* resulted in increased cell density and a slight increase in butanol tolerance. A metabolic network analysis of these DEGs revealed that six genes (*fadA*, *fadB*, *fadD*, *fadL*, *poxB*, and *acs*) associated with acetyl-CoA production were significantly upregulated in DT*rob*, suggesting that Rob inactivation might enhance butanol tolerance by increasing acetyl-CoA. Interestingly, DT*rob* produced more acetate in response to butanol stress than the wild-type strain, resulting in the upregulation expression of some genes involved in acetate metabolism. Altogether, the results of this study reveal the mechanism underlying increased butanol tolerance in *E. coli* regulated by Rob inactivation.

## Introduction

Butanol has received increased attention as a high-energy-density fuel and bulk chemical feedstock (Gu et al., 2011). Only biobutanol can be used in the pharmaceutical and cosmetics industries, and biomass-based butanol production can reduce environmental pollution compared to petrochemical-based production (Jiang et al., 2015b). Traditional biobutanol is produced by *Clostridium* species and can reach a titer of approximately 20 g/L (Qureshi and Blaschek, 2000). The butanol titers produced by engineered *Escherichia coli* strains can also reach approximately 20 g/L in batch fermentation without any antibiotics or inducers and up to 30 g/L with the continuous removal of butanol (Shen et al., 2011; Jang and Lee, 2015; Dong et al., 2017), indicating that *E. coli* is a potential butanol-producing bacterium. However, the current production of butanol is limited to approximately 20 g/L due to the toxicity of butanol toward bacteria; indeed, this toxicity is a bottleneck in butanol production (Qureshi and Blaschek, 2000). Therefore, it is important to improve the butanol tolerance of chassis strains and to explore tolerance-associated mechanisms to promote the highly efficient production of butanol.

Due to its hydrophobicity, butanol binds to lipid chains of the bacterial cytoplasmic membrane, which results in toxicity to the cells (Jones and Woods, 1986; Nielsen and Prather, 2009). The mechanism of this toxicity involves increased membrane fluidity and permeability in the presence of butanol, causing leakage of protons and ATP and interference with the correct folding of proteins, which leads to cell damage or even death (Sikkema et al., 1995; Isken and de Bont, 1998). Bacteria alter their physiological and biochemical characteristics to respond to butanol stress in the following ways: (1) membrane composition changes, including increasing efflux capacity of toxins, preventing leakage of intracellular components, and protecting cells from damage due to the solvent (Reyes et al., 2011; Bui et al., 2015; Royce et al., 2015; Sandoval and Papoutsakis, 2016); (2) physiological responses similar to those involved in responses to osmotic, oxidizing, respiratory, and heat shock stresses (e.g., altered osmotic pressure) (Purvis et al., 2005; Chin et al., 2017), reactive oxygen species (ROS) accumulation (Kaczmarzyk et al., 2014) and enhanced metabolic transport and molecular chaperone levels (Peralta-Yahya et al., 2012); and (3) up- or downregulation of expression of regulatory genes, such as those encoding sensor proteins, transcription factors (TFs), and those involved in regulating expression of small RNAs (Peralta-Yahya et al., 2012), to modulate gene expression profiles to protect against butanol stress (Reyes et al., 2011, 2012).

*Escherichia coli* expresses 304 TFs (Perez-Rueda et al., 2015), though only a few have been reported to regulate butanol tolerance-related genes (Reyes et al., 2012; Horinouchi et al., 2018). Furthermore, the corresponding genes regulated by these TFs and the associated regulatory mechanism have not yet been clarified (Aquino et al., 2017), limiting the improvement of butanol-tolerant chassis strains using a rational-design engineering strategy. In our previous study, a mutant strain (BW1847) able to tolerate 2% (v/v) butanol was obtained, and among the genes mutated or deleted, *rob* (GenBank No. RS22900), *acrB* (GenBank No. RS02385), and *tqs*A (GenBank No. RS08380) have been identified as having a function in enhancing butanol tolerance (He et al., 2019). The DT*rob* strain, with an AT_686__–__7_ base deletion within *rob* in the genome, and the *rob*-deletion mutant Δ*rob* produced much higher cell densities than did the wild-type strain under 0.75% butanol stress (He et al., 2019), indicating that partial and full inactivation of Rob both result in improved butanol tolerance. The *rob* gene encodes a right oriC-binding transcriptional activator (Nakajima et al., 1995) that interacts with a superoxide response regulon transcriptional activator (SoxS) and a multiple antibiotic resistance transcriptional regulator (MarA) and can activate genes involved in antibiotic, oxygen pressure and organic solvent resistance (Nakajima et al., 1995). The *rob*-deleted strain exhibited decreased tolerance to antibiotics, oxygen stress, cyclohexane and n-pentane (Nakajima et al., 1995; White et al., 1997; Bennik et al., 2000). However, inactivation of Rob yielded increased butanol tolerance in our previous study. These contrasting results are due to the extensive, intricate and multiple regulatory mechanisms of Rob, and the corresponding butanol-tolerant mechanism regulated by Rob is unknown. Strain DT*rob* with truncated Rob exhibited a butanol tolerance characteristic and can be used to investigate the interaction mechanism between Rob and its target genes. DT*rob* is thus an ideal candidate for studying the butanol tolerance mechanism caused by Rob inactivation.

Therefore, in this study, butanol stress response genes regulated by the Rob-inactivated mutant were evaluated by RNA-Seq. In addition, key genes involved in butanol tolerance were functionally identified, and a potential tolerance mechanism was determined to demonstrate the novel roles of Rob in response to butanol stress.

## Materials and Methods

### Growth Assays and Extracellular n-Butanol Measurements

The site-specific mutant DT*rob* (AT_686__–__7_ deletion in the *rob* gene) was constructed in a previous study (He et al., 2019). The DT*rob* strain and BW25113 (Supplementary Table S1) were precultured in lysogeny broth (LB) until the late exponential phase, after which the cultures were concentrated to an OD_600_ of 20. The concentrated cells were then used to inoculate 50 mL of LB medium containing 0, 0.75, 1, and 1.25% (v/v) butanol at an initial OD_600_ of 0.1–0.15 to evaluate cell growth. A total of 0.7 mL of culture was taken to measure the OD_600_ with a Cary 50 Conc spectrophotometer (Varian, Palo Alto, CA, United States); 1 mL of culture was collected at the appropriate time to measure butanol and acetate concentrations in the medium. The collected samples were centrifuged, and the supernatants were used to measure butanol and acetate contents by high-performance liquid chromatography (HPLC). HPLC was performed using an organic acid analytical column (Aminex HPX-87H Ion Exclusion Column, 300 mm × 7.8 mm) at 45°C; sulfuric acid (4 mM) was used as the mobile phase at 0.8 mL/min. The butanol and acetate concentrations in each sample were calculated by comparisons with the peak area of the standard. The per unit intracellular butanol concentration (PIC) was calculated according to previous reports (He et al., 2019), with a slight modification, as shown by the following equation:

$$\text{PIC}=\frac{{\text{C}}_{\text{initial}}-{\text{C}}_{\text{final}}}{\text{N}}$$

where “PIC” indicates “per unit intracellular butanol concentration” (μg/L) and “C_*initial*_” and “C_*final*_” indicate “initial extracellular butanol concentration” and “final extracellular butanol concentration”, respectively; “N” indicates the number of cells; and 1OD cells at 600 nm corresponds to 8.3 × 10^8^ cells mL^–1^. N = OD_600_ × 8.3 × 10^8^ mL^–1^. The values of the mean and standard deviation are plotted using the bar and error bar.

Overexpression strains (Supplementary Table S1) were precultured in LB until the late exponential phase, and the cultures were concentrated to an OD_600_ of 20. The concentrated cells were then used to inoculate LB medium containing 0.2% (w/v) L-arabinose, 100 μg/mL ampicillin, and a gradient concentration of butanol at an initial OD_600_ = 0.2. A total of 1 mL of culture was collected at a suitable time point for cell concentration measurements.

### RNA-Seq and Data Processing

The strains BW25113 and DT*rob* were cultured in 50 mL of LB containing 0.75% (v/v) butanol until the OD_600_ reached 0.6–1.1; 3-mL aliquots were harvested, and the cells were washed with ice-cold PBS (phosphate buffer saline) buffer. Total RNA was extracted with TRIzol reagent (Invitrogen, Carlsbad, CA, United States) according to the manufacturer’s instructions. The quality of the total RNA extracted was assessed spectrophotometrically at 230, 260, and 280 nm. The RNA sample with an OD_260_/OD_230_ ratio higher than 1.8 and an OD_260_/OD_280_ ratio between 1.8 and 2.1 was considered as pure and used to cDNA library construction. cDNA library preparation and sequencing were conducted by the Allwegene Technology Company in Beijing, China. Individual libraries were sequenced using the Illumina HiSeq 4000 platform (Illumina, Inc., San Diego, CA, United States). The original fluorescent images were converted into raw sequence reads by CASAVA software. The raw reads were then processed to remove reads with adapters, reads containing poly-N (N > 1%) and low-quality reads (more than 50% bases with Q-score ≤20), yielding clean reads (Gao et al., 2018; Wang et al., 2018). The clean data were deposited in Gene Expression Omnibus (GEO) at NCBI (Accession Number GSE120032) and mapped to the genome of the reference strain BW25113 (LOCUS NZ_CP009273) using Bowtie2 (Kim et al., 2018). Gene expression levels were analyzed using fragments per kilobase of exon model per million mapped reads (FPKM). Differentially expressed genes (DEGs) between the two samples were identified using the DESeq package (v1.24.0), and | log2(FoldChange)| > 1 and q-value < 0.005 were used as the threshold to identify significant differences in gene expression. Analysis of Gene Ontology (GO), which assigns genes into functional categories, was performed using the GOSeq (v1.22) and top GO (v2.22) R packages.

### Quantitative Reverse Transcription PCR Analysis

The expression levels of eighteen DEGs representing different functional categories were assessed by real-time quantitative reverse transcription PCR (qRT-PCR) to validate the reliability of the RNA-Seq data. Total RNA from DT*rob* and BW25113 cells was extracted as described above, and RQ1 RNase-Free DNase (Promega, Madison, WI, United States) was added to the RNA to remove genomic DNA. PCR reaction was performed using the total RNA as template in order to check for genomic DNA contamination. No product was observed, demonstrating that the sample had no genomic DNA contaminants and was suitable for qRT-PCR assay. Subsequently, reverse transcription was performed using iScript cDNA Synthesis Kit (Bio-Rad) with 2 μg of total RNA following the manufacturer’s instructions. qRT-PCR experiments were performed using CFX96 Real-Time System (Bio-Rad). Each reaction contained 2 μL of diluted (1/10) cDNA, Taq SYBR Green qPCR Premix (Yugong Biolabs Inc., Jiangsu, China) and the corresponding primer pairs (Supplementary Table S2). The 16S rRNAgene was used as a reference to normalize the qRT-PCR data. The expression level of each gene was calculated according to the following formula: expression level = 2^–ΔΔ*Ct*^ (Livak and Schmittgen, 2001). The relative mRNA level is presented as the percent (%) ratio of the gene expression level in DT*rob* to that in BW25113.

### Construction of Knockout Strains

Strains deleted for the *glgS* and *yibT* genes were constructed using the CRISPR-Cas9 system (Ran et al., 2013; Jiang et al., 2015a; Supplementary Figure S1). Inverse PCR was performed using pTargetF plasmid as a template to introduce the target sequence of N20 (20-bp complementary region) upstream of the single-guide RNA (sgRNA) in the pTargetF plasmid. The N20 sequence was introduced into the primers shown in Supplementary Table S3. The inverse PCR products were digested with the methylation-sensitive restriction enzyme DMT (TransGen Biotech, Shanghai, China) to remove methylated plasmid templates, after which the DNA was transformed into DH5α competent cells (Biomed, Beijing, China). Positive clones were identified by PCR with the appropriate geneN20F/pTargetF-IR primer pairs (Supplementary Table S3), and the resulting plasmid was correspondingly named pTargetF-geneN20. DNA fragments containing left and right homologous arms located upstream and downstream of the target gene were amplified with the primer pairs geneDLF/geneDLR and geneDRF/geneDRR (Supplementary Table S3), respectively. The two fragments were then fused together by overlap PCR with the appropriate geneDLF/geneDRR primer pairs (Supplementary Table S3), and the resulting fused PCR fragment was used as donor DNA to delete the target gene by homologous recombination. Approximately 500 ng of donor DNA and the corresponding pTargetF-geneN20 plasmid were co-transformed into BW25113 (pCas) competent cells. Positive clones were screened on LB plates containing 100 mg/L kanamycin and spectinomycin and subsequently identified by PCR using the appropriate geneDLF/geneDRR primer pairs (Supplementary Table S3). The deletion was confirmed by sequencing, and the positive clones were cultured in LB medium supplemented with 50 mg/L kanamycin and 0.5 mM IPTG to eliminate the pTargetF-geneN20 plasmid. The temperature-sensitive plasmid pCas was then removed by growing the culture overnight at 37°C.

### Overexpression Analysis of Several Upregulated Genes

Thirteen upregulated genes (Supplementary Table S5) were chosen for overexpression analysis. Plasmids harboring the target genes were constructed on the basis of pBAD30 (Guzman et al., 1995). PCR amplification of the target genes from the genomic DNA of BW25113 was performed using the primer pairs shown in Supplementary Table S4. The plasmid pBAD30 was digested with the restriction endonucleases *Sac*I and *Hin*dIII, and the aforementioned PCR products were cloned into the *Sac*I/*Hin*dIII sites of pBAD30 using GenBuilder Cloning Kit (GenScript, Nanjing, China). The mixture was transformed into competent DH5α cells and screened on LB agar plates containing 100 μg/mL ampicillin. Single clones were identified by PCR using the primers pBADIup/pBADIdown (Supplementary Table S4). Plasmids were extracted using TIANprep Mini Plasmid Kit (TIANGEN, Beijing, China) and verified by sequencing (Sangon Biotech, Shanghai, China). The overexpression plasmids were transformed into BW25113 by electroporation, and the positive transformants were correspondingly named BW25113 (gene) (Supplementary Table S1).

## Results

### Enhanced Butanol Tolerance by the Truncated Rob in DT*rob*

**FIGURE 1:**
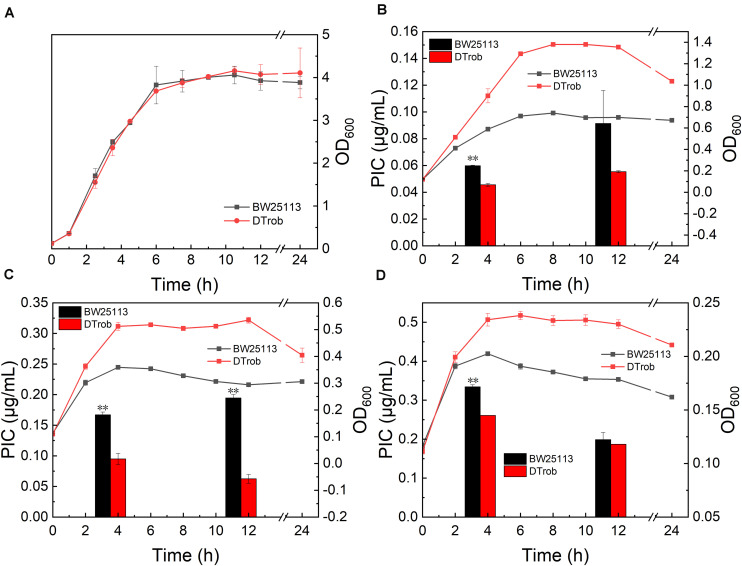
Growth assays for the DT*rob* strain in the presence of butanol. **(A)** Without butanol; **(B)** with 0.75% (v/v) butanol; **(C)** with 1% butanol; and **(D)** with 1.25% butanol. The line graph shows the growth curve, and the bar graph shows the unit intracellular butanol concentration. Error bars represent one standard deviation. Statistical significance of the differences between DT*rob* and BW25113 was analyzed by a one-way analysis of variance (ANOVA). *P < 0.05 was considered statistically significant and **p < 0.01 was considered extremely significant. Growth assays were performed in triplicate in screw-cap flasks.

The DT*rob* strain has an AT_686__–__7_ base deletion of *rob* in the genome, which leads to an early termination of translation of *rob* mRNA, and the resulting truncated protein only has 229 amino acids (He et al., 2019). Strain DT*rob* showed a similar growth trend to control BW25113 in absence of butanol (Figure 1A), and the maximum cell density of the DT*rob* exhibit 94, 49, and 18% higher than that of BW25113 under 0.75, 1, and 1.25% (v/v) butanol stress, respectively, indicating that the DT*rob* strain was able to tolerate 1–1.25% and that the improvement in relative growth decreased with increasing butanol concentrations. The PIC of the DT*rob* strain was 76, 56, and 78% of that of the control BW25113 under 0.75, 1, and 1.25% (v/v) butanol stress at 4 h (logarithmic phase), respectively (Figures 1B–D), which shows that inactivation of Rob can result in an enhanced ability to efflux butanol out of the cell to improve the tolerance, and the highest efflux capacity was shown in the presence of 1% butanol. The PIC value of DT*rob* was 32–60% of that of BW25113 in 0.75–1% (v/v) butanol at 12 h, demonstrating a higher efflux capacity than at 4 h. Nevertheless, interestingly, the PICs of both BW25113 and DT*rob* in a higher concentration of butanol (1.25%) were decreased at 12 h compared to 4 h, and the two strains had similar intracellular butanol concentration. It is due to that the strains grown in 1.25% butanol have entered the decline phase at 12 h (Figure 1D), and the cell death causes more butanol to be released into the medium, thus resulting in a decreased sharply butanol content in these cells and lower PIC value than at 4 h. Therefore, the alleviated toxicity by butanol efflux in log stage is one reason for the improved butanol tolerance of DT*rob*.

### Transcriptomic Differences Between the DT*rob* and Control Strains Under Butanol Stress

RNA-Seq of the wild-type BW25113 and DT*rob* strains was performed to assess differences in gene expression between them. Genes with a “q-value < 0.005” and “| log2(FoldChange)| > 1” were defined as DEGs. A total of 285 (6.2% of genes) DEGs were identified in DT*rob* compared with the control. In the late logarithmic phase, 184 and 101 genes were upregulated and downregulated in DT*rob* compared with BW25113, respectively (Supplementary Figure S2A). The 285 DEGs were then subjected to GO enrichment analysis and grouped into three primary categories: biological process, molecular function and cellular component. GO annotations were found for 202 of these genes. The significantly enriched GO terms and number of DEGs with GO annotations were as follows: “localization” (32.7%, 66/202), “transport” (31.7%, 64/202), “establishment of localization” (31.7%, 64/202), “oxidoreductase activity” (17.82%, 36/202), and “transporter activity” (20.30%, 41/202) (Supplementary Figure S2B). In addition, the 285 DEGs were examined by Kyoto Encyclopedia of Genes and Genomes (KEGG) pathway analysis using KOBAS (v2.0), revealing 68 pathways, 53 of which were different from the KEGG pathways in previous reports (Reyes et al., 2012; Si et al., 2016; Guo et al., 2019). The significant KEGG terms are primarily associated with the biosynthesis of antibiotics, biosynthesis of secondary metabolites and fatty acid degradation (Supplementary Figure S3).

Hundreds of potential genes involved in the n-butanol tolerance response have been identified by DNA microarrays and comparative genome hybridization microarrays (array-CGH); nevertheless, only approximately 20 candidate genes were identified as being associated with butanol tolerance through overexpression or knockout experiments (Supplementary Table S6; Rutherford et al., 2010; Reyes et al., 2011; Si et al., 2016). Among the 285 DEGs identified by RNA-Seq in the present study, *ompF*, *acrB*, *glcF*, *ybjC*, *yibT* and *cpxP* have been reported to be associated with butanol tolerance (Rutherford et al., 2010; Reyes et al., 2011; Si et al., 2016); however, most of the DEGs have not yet been reported to be involved in butanol tolerance. Therefore, the results of this study reveal potential functional genes related to the development of butanol-tolerant strains for further exploration of butanol tolerance mechanisms.

### Validation of RNA-Seq Data Using Real-Time qRT-PCR

Twenty five genes representing different GO functional categories and different expression levels were classified based on annotations in the NCBI database (Table 1) and primarily classified into four groups: regulatory factors (ChaC, GadE, GlgS, and YiaG), transport and membrane proteins (ActP, ElaB, PspG, FadL, MdtD, OmpW, Slp, YacH, YibT, YdcL, and YjcH), stress response proteins (HdeB, InaA, and YhbO), and enzymes (Acs, AstC, NarG, FadA, FadB, FadD, and PoxB). The expression levels of 25 DEGs between the DT*rob* and BW25113 strains under butanol stress were analyzed by qRT-PCR to confirm the reliability of the RNA-Seq data obtained. The log2-fold change values of the 25 DEGs were also examined to compare the consistency of the RNA-Seq and qRT-PCR data, and a strong correlation (*R* = 0.8318) was observed between the RNA-Seq and qRT-PCR data (Supplementary Figure S4). Based on the qRT-PCR results, the expression trends of 23 of these candidate genes were consistent with the RNA-Seq data. Among these 23 genes, *acs*, *actP*, *astC*, *elaB*, *fadA*, *fadB*, *fadD*, *fadL*, *yiaG*, *yhbO*, and *yjcH* were obviously upregulated (263–11503%) (Table 1) and *gadE*, *hdeB*, and *slp* slightly upregulated (153–194%) in the DT*rob* strain compared to the control strain, whereas *chaC*, *glgS*, *inaA*, *narG*, *mdtD*, *yibT*, *ydcL*, and *yacH* were notably downregulated (65–99%) (Table 1). Therefore, 92% of the candidate genes exhibited consistent qRT-PCR and RNA-Seq results, indicating that the RNA-Seq data were of good quality.

**TABLE 1:** Functional categories and qRT-PCR results of 25 DEGs associated with butanol tolerance.

**Functional group and gene**	**Description**	**Fold change^a^**	**Relative expression by qRT-PCR^b^ (%)**	**True or false^c^**
Regulator				
*gadE*	Transcriptional regulator GadE	2.68	153	True
*chaC*	Cation transport regulator	−2.65	30	True
*glgS*	Glycogen synthase surface composition regulator/motility and biofilm regulator	−5.83	1	True
*yiaG*	Transcriptional regulator	2.03	4095	True
Stress related				
*hdeB*	Acid stress chaperone HdeB	3.23	181	True
*inaA*	Acid-inducible Kdo/WaaP family putative kinase	−3.06	8	True
*yhbO*	Stress-resistance protein	2.38	263	True
Metabolism				
*narG*	Respiratory nitrate reductase 1 alpha chain	−3.55	27	True
*acs*	Acetyl-coenzyme A synthetase	3.33	3000	True
*astc*	Succinylornithine aminotransferase	3.98	3928	True
*fadA*	Acetyl-CoA C-acyltransferase FadA	3.63	2508	True
*fadB*	Fatty acid oxidation complex subunit alpha FadB	3.27	4580	True
*fadD*	Acyl-CoA synthetase	2.14	1068	True
*poxB*	Pyruvate dehydrogenase	1.36	5686	True
Membrane and transport related				
*pspG*	Envelope stress response protein PspG	−2.7	124	False
*elaB*	DUF883 family protein, putative membrane-anchored ribosome-binding protein	2.19	11503	True
*fadL*	Long-chain fatty acid transporter	2.81	7286	True
*actP*	Cation acetate symporter	3.11	797	True
*mdtD*	MFS (major facilitator superfamily) transporter	−3.06	23	True
*yibT*	Uncharacterized protein Hypothetical protein	−5.76	2	True
*yjcH*	DUF485 family inner membrane protein	3.01	736	True
*slp*	Outer membrane protein slp	2.80	194	True
*ydcL*	Lipoprotein	−2.36	13	True
*ompW*	Outer membrane protein W	3.03	64	False
*yacH*	DUF3300 domain-containing protein	−4.06	35	True

*^*a*^Fold change indicates the log2 ratios between the corresponding transcript levels of the DT*rob* and BW25113 strains in the RNA-Seq data; a log2 fold change of “+” indicates that the gene is upregulated in the DT*rob* strain relative to the BW25113 strain, and a “−” indicates that a gene is downregulated. ^*b*^The percentage ratio of the expression levels of the target gene in the DT*rob* strain compared to that observed in the BW25113 strain. ^*c*^“True” and “False” indicate that the real-time qRT-PCR verification result is consistent and inconsistent with the RNA-Seq data, respectively.*

### Functional Analysis of Significant Differentially Expressed Genes

**FIGURE 2:**
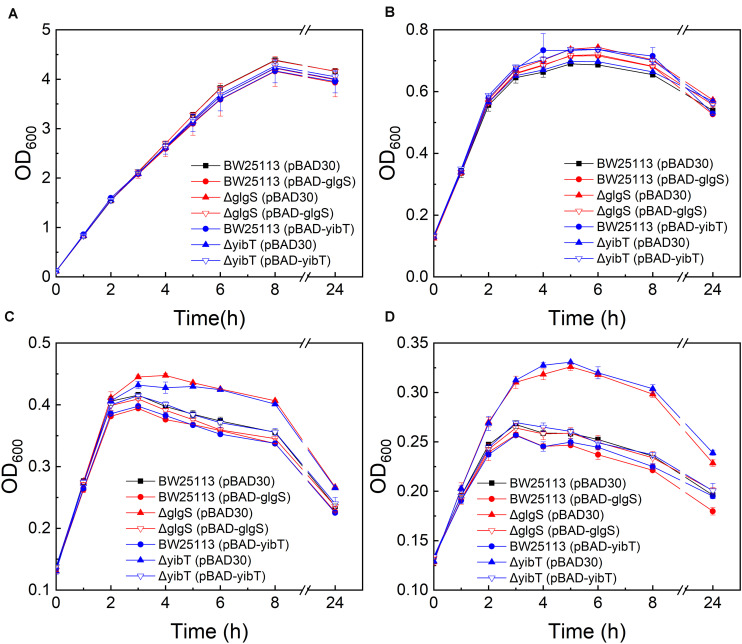
Butanol tolerance evaluation of the *glgS* or *yibT* deletion and complement strains. All strains were cultured in LB medium containing 0% **(A)**, 0.75% **(B)**, 1% **(C)**, and 1.25% (v/v) **(D)** butanol.

Based on GO enrichment and the gene expression levels of the DEGs, fifteen representative DEGs in each cluster were selected (Supplementary Table S5) for investigation of their function in promoting butanol tolerance by growth evaluation under butanol stress. To determine their direct impacts on the butanol tolerance phenotype, the selected upregulated and downregulated genes were overexpressed and deleted, respectively. Deletion mutants of *glgS* and *yibT* were obtained using the CRISPR-Cas9 system and named Δ*glgS* and Δ*yibT*, respectively. The growth evaluation results showed that the maximum cell density of the Δ*glgS* (pBAD30) and Δ*yibT* (pBAD30) strains was increased by 7.7–23.6% compared to that of BW25113 in the presence of 1–1.25% (v/v) n-butanol, indicating that the absence of Rob or downregulated expression improved butanol tolerance (Figure 2). The complementation strains Δ*glgS* (pBAD-*glgS*) and Δ*yibT* (pBAD-*yibT*) of the two genes displayed a lower cell density than their deletion strains Δ*glgS* and Δ*yibT*, indicating that their overexpression can lead to a decrease in butanol tolerance (Figures 2C,D). Overexpression of these two genes in the wild-type strain also reduced butanol tolerance compared to the control strain BW25113 (pBAD30) (Figure 2D). These results indicate that a reduced level of *glgS* and *yibT* expression is beneficial for enhancing butanol tolerance.

**FIGURE 3:**
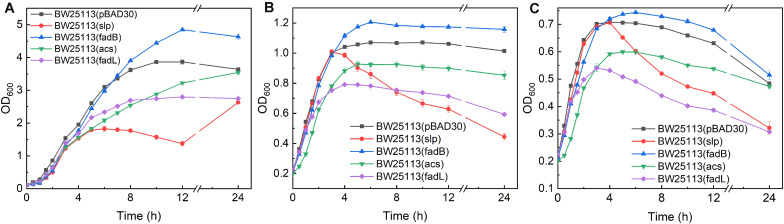
Growth evaluation of overexpression strains. **(A–C)** Growth curves of *acs*-, *fadB*-, *fadL*-, and *slp*-overexpressing strains exposed to 0, 0.5, and 0.75% (v/v) butanol. BW25113 (pBAD30) was used as a control.

Thirteen upregulated genes verified by qRT-PCR were selected for investigating their function in promoting butanol tolerance, and engineered strains were cultured in the presence or absence of butanol. The maximum cell density of BW25113 (pBAD-*fadB*) was increased by 25.5, 12.7, and 5.1% under 0, 0.5, and 0.75% (v/v) butanol stress, respectively, compared with that of the control strain BW25113 (pBAD30) (Figure 3). These results indicate that *fadB* overexpression can promote cell growth and that the cell density of *E. coli* under butanol stress can also be increased to some extent. The maximum cell density of the *slp*-, *acs*-, and *fadL*-overexpressing strains was decreased by 31.9, 8.3, and 27.7%, respectively, compared with the control strain in the absence of butanol (Figure 3A, with a significant decrease under butanol stress (Figures 3B,C). These results indicate that overexpression of these three genes inhibits the growth of the cells, resulting in a decreased cell density under butanol stress.

Unlike *fadB*, *slp*, *acs*, and *fadL*, overexpression of the other nine upregulated genes did not cause an increase or decrease in cell growth under butanol stress (data not shown), indicating that overexpression of these nine genes does not alter butanol tolerance. It is possible that most responsive genes do not exhibit a significant butanol tolerance function; it also may be that simultaneous changes in the expression of multiple DEGs cause synergistic physiological and biochemical responses to resist butanol stress. Therefore, the function and classification of the DEGs is not well illustrated merely by functional complementation via knockout or overexpression of a certain gene. Correspondingly, the metabolic network of these DEGs was also analyzed, and the following interesting response mechanism was observed.

### DT*rob* Responds to Butanol Stress by Altering Acetyl-CoA and Acetate Production

**FIGURE 4:**
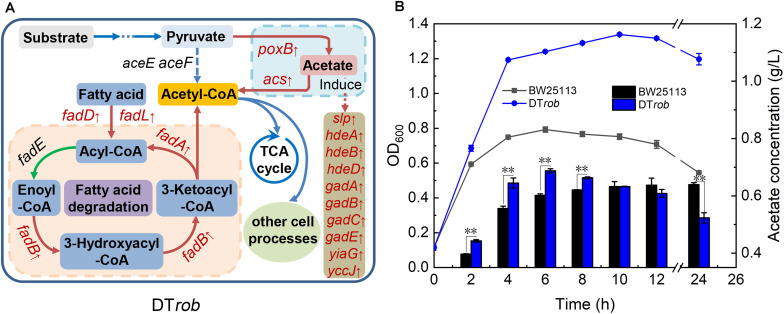
Mechanism of butanol tolerance regulated by Rob via an increase in acetyl-CoA. **(A)** The fatty acid oxidation and acetate metabolism pathways regulated by Rob. Genes marked in red font were upregulated in DT*rob*. **(B)** Acetate levels in the BW25113 and DT*rob* strains under 0.75% (v/v) butanol stress. The line graph shows the growth curve, and the bar graph shows the extracellular acetate concentration. Error bars represent one standard deviation. Statistical significance of the differences between DT*rob* and BW25113 was analyzed by a one-way ANOVA. *P < 0.05 was considered statistically significant and **p < 0.01 was considered extremely significant. Growth assays were carried out in triplicate in screw-cap flasks.

**FIGURE 5:**
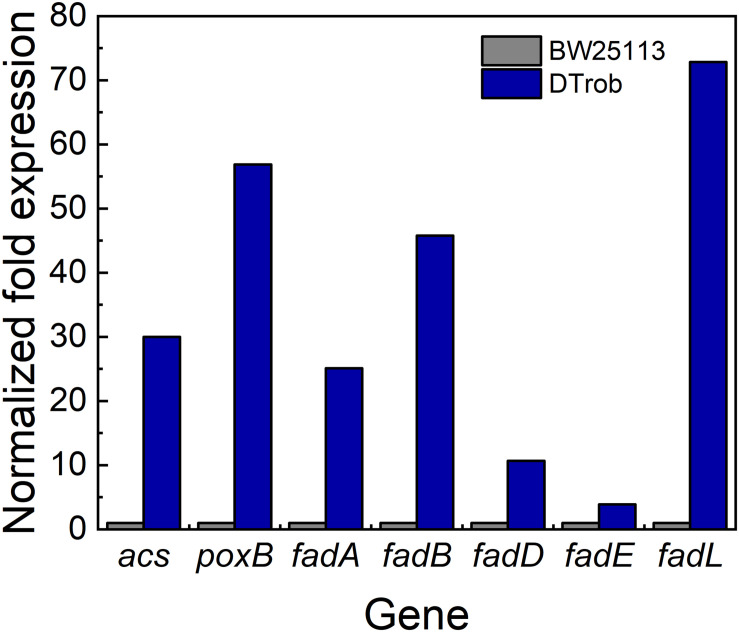
qRT-PCR analysis of genes involved in acetyl-CoA production. Gray bar represents BW25113, blue bar represents DT*rob*. The expression level was calculated by 2^–ΔΔ*Ct*^ method.

The expression levels of *poxB*, encoding pyruvate dehydrogenase (PoxB), and *acs*, encoding acetyl-CoA synthetase (Acs), were upregulated in the DT*rob* strain, as shown by the RNA-Seq and qRT-PCR data (Figure 5). PoxB and Acs convert pyruvate to acetyl-CoA in a two-step enzymatic reaction (Figure 4A). Four genes (*fadA*, *fadB*, *fadD*, and *fadL*) involved in fatty acid β-oxidation were also significantly upregulated in the DT*rob* strain, and the final product of fatty acid β-oxidation is acetyl-CoA (Figure 4A). An enhanced acetyl-CoA pool has been demonstrated to be achieved through modification of a fatty acid β-oxidation pathway (Zhang et al., 2019), and acetyl-CoA is a key molecule in microbial central carbon metabolism and involved in a variety of cellular processes (Krivoruchko et al., 2015). Thus, upregulation of the six genes (Figure 5 and Table 1) caused by inactivation of Rob might result in increased acetyl-CoA levels, which would provide more raw material and energy for a number of physiological processes and cell growth. Nonetheless, the acetyl-CoA content of BW25113 and DT*rob* was not significantly different under 0.75% butanol stress (data not shown). It is possible that the increased acetyl-CoA in DT*rob* is quickly transferred to other metabolic pathways, such as the TCA cycle to provide energy for cell growth, or provides acetyl groups for acetylation of histones to promote cell growth and proliferation (Cai et al., 2011). Accordingly, no obvious accumulation of acetyl-CoA was observed in DT*rob*, although which showed a higher cell density than BW25113 under butanol stress.

In addition, expression of ten genes (*Slp*, *hdeA*, *hdeB*, *hdeD*, *gadA*, *gadB*, *gadC*, *gadE*, *yiaG*, and *yccJ*) upregulated in the DT*rob* strain has been reported to be increased when cells are subjected to acid stress (Ma et al., 2003; Tucker et al., 2003; Hommais et al., 2004). Indeed, upregulation of PoxB, which converts pyruvate to acetate (Dittrich et al., 2005), would lead to an increase in acetate levels, and the extracellular acetate concentration in the DT*rob* culture was higher than that of the BW25113 strain in the log phase (Figure 4B). These results indicate that upregulated expression of some genes in the DT*rob* strain might promote the production of acetyl-CoA and result in acetate accumulation, in turn inducing expression of genes involved in the response to acid stress.

## Discussion

Mutation of the transcription regulator *rob* in *E. coli* altered the expression levels of 285 genes in response to butanol stress. Deletion of the genes *glgS* and *yibT*, encoding a transcriptional regulator and a membrane-associated protein, respectively, directly increased butanol tolerance. In addition, overexpression of the *fadB* gene, encoding a fatty acid oxidation-related enzyme, promoted the growth of *E. coli* and slightly enhanced butanol tolerance. Deletion of *glgS* has been reported to improve the production of both flagella and type 1 fimbriae, resulting in enhanced swarming motility of bacterial cells. Such enhanced motility protects cells from toxicity by allowing them to escape from toxic environments, such as those containing n-butanol (Rahimpour et al., 2013; Kim et al., 2016). However, the Δ*glgS* strain showed an increased capacity to initiate biofilm formation (Rahimpour et al., 2013), and biofilms provide good protection for cells as a permeability barrier and also enhance the ability of bacteria to adapt to adverse environments, including butanol stress (Li et al., 2016). It has been reported that deletion of *yibT* can increase the proportion of unsaturated fatty acids in the membrane, resulting in lower membrane fluidity and higher membrane rigidity and integrity, protecting cells from organic solvent stress (Si et al., 2016). Therefore, *yibT* deletion can improve the butanol tolerance of *E. coli*.

The gene *fadB* encodes the α-subunit of a multienzyme complex that is involved in fatty acid β-oxidation (Fujita et al., 2007) and responsible for the hydration of enoyl-CoA and oxidation of 3-hydroxyacyl-CoA (Figure 4A). Overexpression of *fadB* may accelerate the two-step enzymatic reaction and the conversion of fatty acids to acetyl-CoA. Additional acetyl-CoA entering the tricarboxylic acid cycle would provide more energy for cell growth, resulting in an increase in cell density. This growth improvement may also result in a higher cell density under butanol stress.

Interestingly, overexpression of *acs*, *slp*, and *fadL* did not increase the butanol tolerance of *E. coli* and even led to inhibition of cell growth. The *acs*, *slp*, and *fadL* genes encode the Acs (Brown et al., 1977), outer membrane protein Slp (Alexander and St John, 1994) and fatty acid transporter FadL (van den Berg et al., 2004), respectively, and the results of previous studies also showed that overexpression of these three genes inhibits cell growth (Black et al., 1985; Alexander and St John, 1994; Zhang et al., 2019). However, individually overexpressing these three genes does not improve butanol tolerance, as they function in synergy with other genes in the same GO functional category to resist butanol stress. Moreover, Acs and PoxB may function together to promote the production of acetyl-CoA; FadL may act in conjunction with proteins encoded by *fadA*, *fadB*, and *fadD* to promote fatty acid degradation, and Slp may act in conjunction with some proteins to promote acid tolerance in response to acetate stress (Figure 4A). Therefore, the proteins encoded by these genes synergistically respond to butanol stress.

Among the 15 DEGs identified by functional complementation experiments, only *fadB*, *glgS*, and *yibT* were identified to be involved in significant butanol tolerance in this study. Correspondingly, among thirteen DEGs investigated, only five contribute to the increased butanol tolerance of the mutant B8 (Si et al., 2016). These results demonstrate that changes in the expression of a few genes may be valuable for functional improvements in butanol tolerance. Furthermore, the enhanced tolerance regulated by a TF may result from the combined actions of simultaneous changes in the expression levels of multiple genes. In addition, metabolic network and GO analyses of DEGs are necessary to identify potential mechanisms regulated by TFs, which would provide a basis for further elucidating the elaborate molecular interplay between a TF and its interacting proteins or responsive genes.

## Conclusion

In summary, the *rob* gene-mutant strain DT*rob* was shown to exhibit higher butanol tolerance than BW25113, and 285 DEGs between the two strains were identified. Deletion of *glgS* and *yibT* and overexpression of *fadB* improved the butanol tolerance of *E. coli*. Furthermore, this is the first study to report that *glgS* can impact butanol tolerance directly. Analysis of the metabolic network of DEGs revealed that inactivation of Rob upregulated some genes that may be involved in the synergistic increase in acetyl-CoA production to promote cell growth under butanol stress. Thus, the results of this study provide a deeper understanding of the regulatory mechanism of Rob in the resistance of *E. coli* to butanol stress.

## Data Availability Statement

The datasets generated for this study can be found in the Gene Expression Omnibus (GEO) of NCBI, GSE120032, https://www.ncbi.nlm.nih.gov/geo/query/acc.cgi?acc=GSE120032.

## Author Contributions

YM conceived of the project, analyzed the data, and wrote the manuscript. YM, TX, and ZW designed the experiments and drafted the manuscript. TX, ZW, and DH performed the experiments. All authors analyzed the data, prepared the manuscript, and approved the final version.

## Conflict of Interest

The authors declare that the research was conducted in the absence of any commercial or financial relationships that could be construed as a potential conflict of interest.
