# Supplementary material for: A Novel Butanol Tolerance-Promoting Function of the Transcription Factor Rob in Escherichia coli
Source: Front Bioeng Biotechnol. 2020 Sep 22;8:524198. doi: 10.3389/fbioe.2020.524198 (PMC7537768; doi:10.3389/fbioe.2020.524198)
Supplement: Supplementary file 1 [file Data_Sheet_1.pdf]

Supplementary Material

1 Supplementary Figures

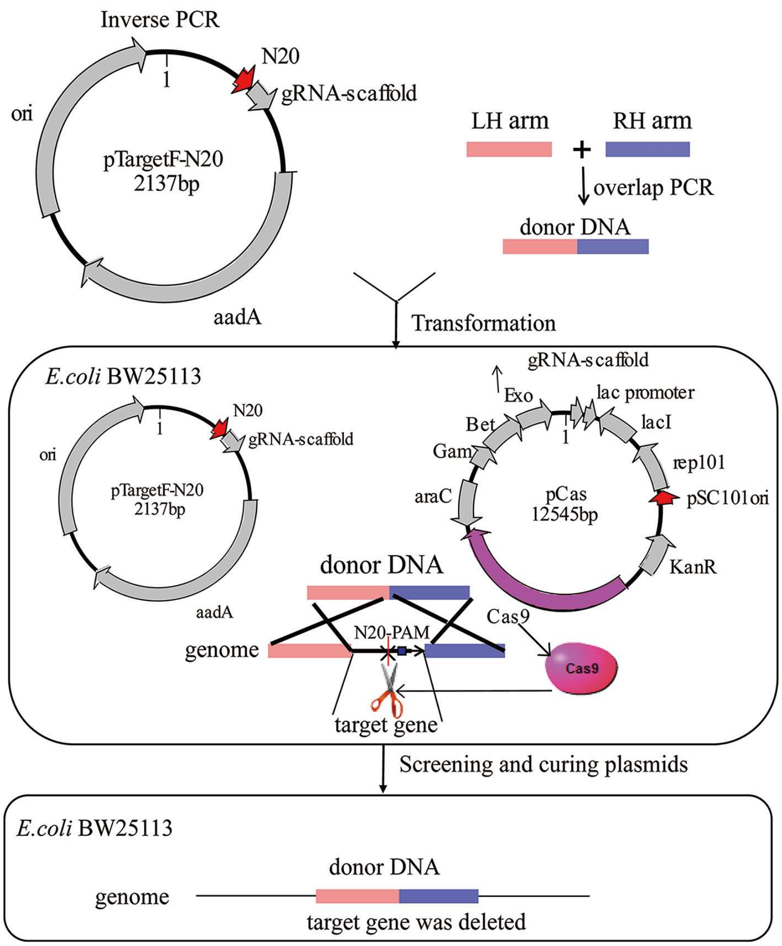

Figure S1. The diagram of genome editing with crispr-Cas9 system(Jiang et al., 2015). pTargetF-N20 was constructed to express the targeting sgRNA, with the pTargetF plasmid as templates by inverse PCR. N20, 20-bp region complementary to the targeting region. Donor DNA were fused from the left and right homologous arms by overlap PCR. pCas plasmid contains the cas9 gene, which express Cas9 endonuclease. AraC, arabinose-inducible transcription factor; KanR: kanamycin resistance expression cassette. The sgRNA-N20 can mediate Cas9(like a molecular scissors) cleavage at ~3 bp upstream of the PAM of the target gene in the genome, and the doner DNA could repair incision by λ-RED recombinase yielding a deletion of target gene. Finally, an arabinose-inducible sgRNA(in pCas plasmid) guiding Cas9 to the pMB1 replicon of pTargetF-N20, the λ-Red recombination system to improve the editing efficiency, and the temperature-sensitive replication repA101 for self-curing.

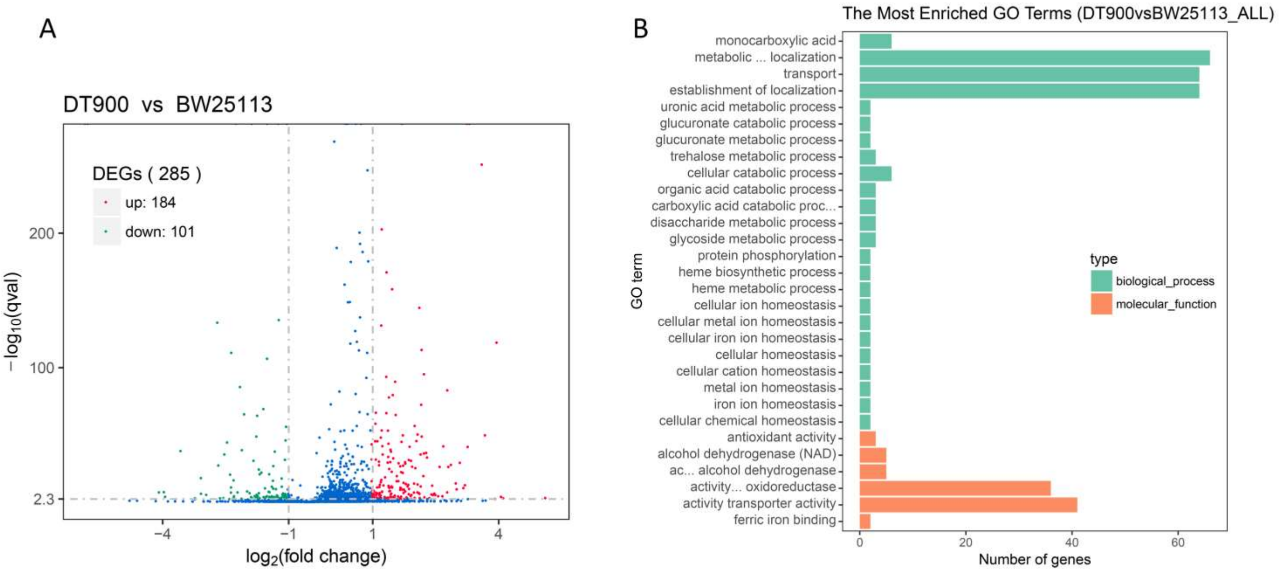

Figure S2. Screening and functional analysis of DEGs between DT*rob* and the control. (A) Significantly up- or downregulated genes between the DT*rob* and BW25113 strains (|log2(FoldChange)| > 1 and q value < 0.005); (B) GO enrichment analysis of the DEGs.

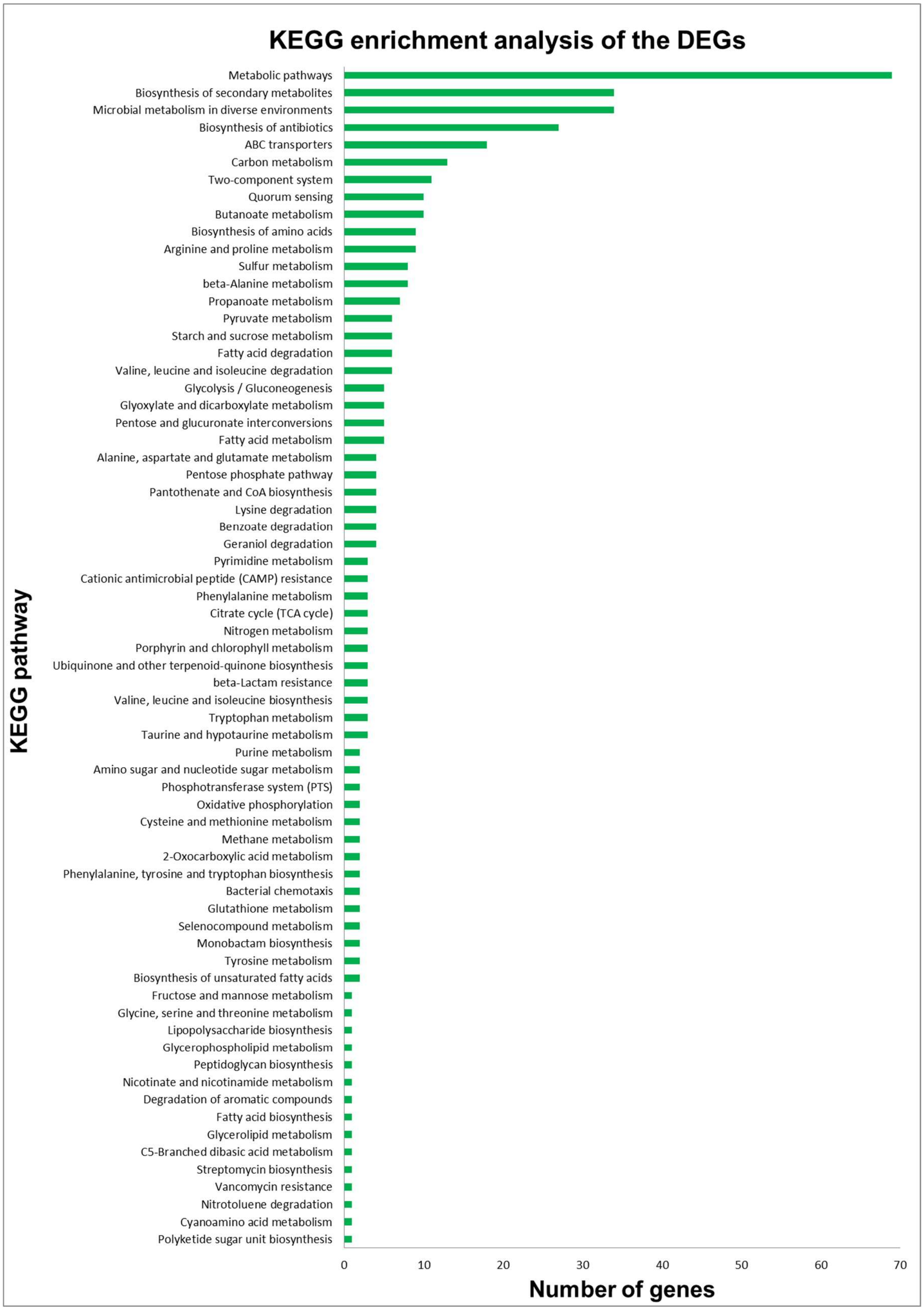

Figure S3. KEGG enrichment analysis of the DEGs.

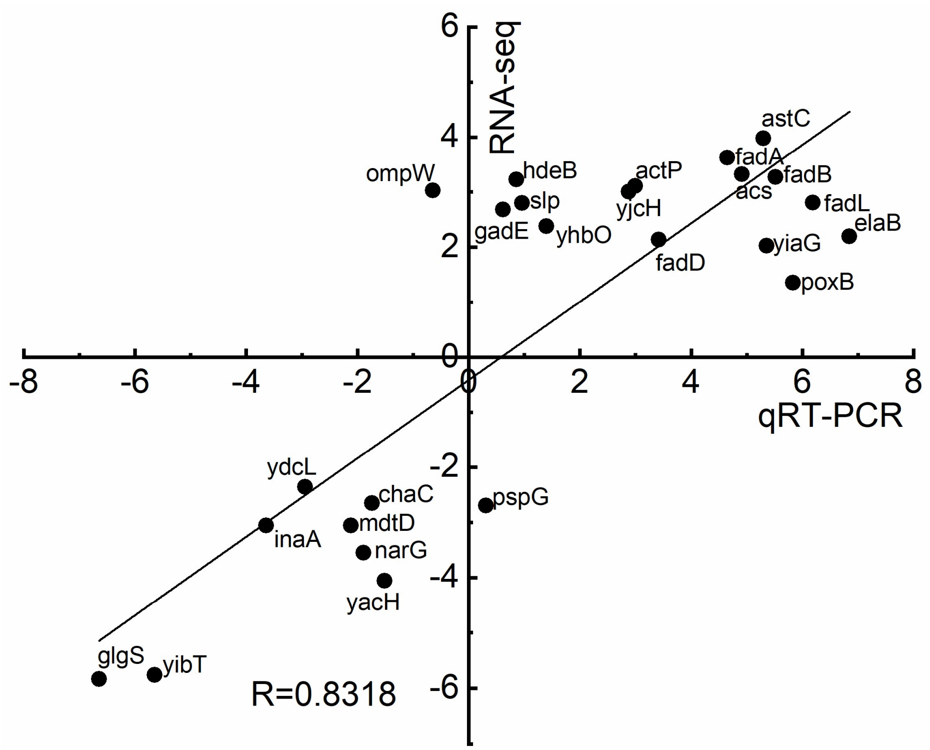

Figure S4. Correlation between RNA-seq and qRT-PCR results for RNA-seq data verification. The gene expression ratios of both RNA-seq and qRT-PCR data for the genes in Table 1 were log transformed into base 2, and the RNA-seq log2 ratio values were plotted against the qRT-PCR log2 values.

2 Supplementary Tables

Table. S1 Strains and plasmids used in this study.

Strains and plasmids	Genotype/relevant characteristics	Source
**Strains**		
E.coli BW25113	F^- *△(araD-araB)567 △lacZ4787(::rrnB-3)λ⁻rph-1 △(rhaD-rhaB)568 hsdR514*	CGSC[a]
Δ*rob*[b]	BW25113 △*rob*::Cm^r	(He et al., 2019)
DT*rob*[b]	*E.coli* BW25113, point mutation of *rob*	This study
Δ *glgS*	BW25113 △*glgS*	This study
Δ *yibT*	BW25113 △*yibT*	This study
BW25113(pBAD30)	BW25113 harboring plasmid pBAD30	This study
BW25113(pBAD-*acs*)	BW25113 harboring plasmid pBAD30-*acs*	This study
BW25113(pBAD-*actP*)	BW25113 harboring plasmid pBAD30-*actP*	This study
BW25113(pBAD-*astC*)	BW25113 harboring plasmid pBAD30-*astC*	This study
BW25113(pBAD-*elaB*)	BW25113 harboring plasmid pBAD30-*elaB*	This study
BW25113(pBAD-*fadA*)	BW25113 harboring plasmid pBAD30-*fadA*	This study
BW25113(pBAD-*fadB*)	BW25113 harboring plasmid pBAD30-*fadB*	This study
BW25113(pBAD-*fadL*)	BW25113 harboring plasmid pBAD30-*fadL*	This study
BW25113(pBAD-*gadE*)	BW25113 harboring plasmid pBAD30-*gadE*	This study
BW25113(pBAD-*hdeB*)	BW25113 harboring plasmid pBAD30-*hdeB*	This study
BW25113(pBAD-*slp*)	BW25113 harboring plasmid pBAD30-*slp*	This study
BW25113(pBAD-*yhbO*)	BW25113 harboring plasmid pBAD30-*yhbO*	This study
BW25113(pBAD-*yiaG*)	BW25113 harboring plasmid pBAD30-*yiaG*	This study
BW25113(pBAD-*yjcH*)	BW25113 harboring plasmid pBAD30-*yjcH*	This study
BW25113(pBAD-*glgS*)	BW25113 harboring plasmid pBAD30-*glgS*	This study
BW25113(pBAD-*yibT*)	BW25113 harboring plasmid pBAD30-*yibT*	This study
**Plasmids**		
pCas	repA101(Ts) kana Pcas-cas9 ParaB-Red lacIq Ptrc-sgRNA-pMB	(Jiang et al., 2015)
pTargetF	pMB1 aadA sgRNA-pMB1	(Jiang et al., 2015)
pKD46	Ap^R, λ Red recombinase expression	(Datsenko and Wanner, 2000)
pBAD30	Arabinose-inducible expression vector, ori 15A replicon, Ap^R	(Guzman et al., 1995)
pKD3	Derived plasmid of pANTS γ , ori R6K replicon， Ap^R, Cm^r	(Datsenko and Wanner, 2000)
pBAD30-*acs*	pBAD30 containing *acs* gene	This study
pBAD30-*actP*	pBAD30 containing *actP* gene	This study
pBAD30-*astC*	pBAD30 containing *astC* gene	This study
pBAD30-*elaB*	pBAD30 containing *elaB* gene	This study
pBAD30-*fadA*	pBAD30 containing *fadA* gene	This study
pBAD30-*fadB*	pBAD30 containing *fadB* gene	This study
pBAD30-*fadL*	pBAD30 containing *fadL* gene	This study
pBAD30-*gadE*	pBAD30 containing *gadE* gene	This study
pBAD30-*hdeB*	pBAD30 containing *hdeB* gene	This study
pBAD30-*slp*	pBAD30 containing *slp* gene	This study
pBAD30-*yhbO*	pBAD30 containing *yhbO* gene	This study
pBAD30-*yiaG*	pBAD30 containing *yiaG* gene	This study
pBAD30-*yjcH*	pBAD30 containing *yjcH* gene	This study
pBAD30-*glgS*	pBAD30 containing *glgS* gene	This study
pBAD30-yibT	pBAD30 containing *yibT* gene	This study

[a]Coli Genetics Stock Center

[b]DT*rob* and Δ*rob* were named DT900 and D900 in our previous study (He et al., 2019), respectively

Table S2. Primers used in real-time quantitative RT-PCR experiment.

Primers	Sequence(5'– 3')
GAPDH-F	GACGAAGTTGGTGTTGAC
GAPDH-R	GATGTGTTTACGAGCAGTT
ompW-F	TTCAGCGTGACCAATAAC
ompW-R	CCAGTAATTCCACACCAAT
inaA-F	CTGGTGACTGAAGATATG
inaA-R	GTCAGAATAAGGCGATAC
glgS-F	GGATGAAGAACATAGAACCT
glgS-R	TCAGTGCTCTAACTCCAG
yibT-F	CGATAAAGCCGTAGATTTC
yibT-R	AAGCCGACGATAATACTC
pspG-F	AATATGTTCCTCGGCGGTAT
pspG-R	CTGATATTTCGGCACTTTTGG
mdtD-F	GAATGAAGCGAATTGTGGTA
mdtD-R	GTAGTCATAAACAACAGGGT
yjcH-F	TATCAGCGGATAGAAGACAA
yjcH-R	GGCGATCAGTAAAATAAAGC
agaD-F	CAAGGATCTGAAGCTGGATA
agaD-R	GTACTGAATACCGCCGTTAT
ydcL-F	TATTGGCTCTGTCTGGTTGT
ydcL-R	CGATGCTGTCATATTTGCTTTG
gadE-F	CCTTGATGAAGAAGCGATTA
gadE-R	GGCAAGTGTTTACCATAAGT
slp-F	GGCAATAACCAACCTGATAT
slp-R	TCCAACGGTAATACAGAGAT
fadB-F	CGTGGATATTGTGGTAGAAG
fadB-R	TGATAGGAATGGTTGAAGTG
hedB-F	CAGTATATAAAGGTGGCGATAC
hedB-R	ATTCGGCAAGTCATTAGATG
chaC-F	GGAACCAACGCACAATACCT
chaC-R	AAATTCTCCGCCAGCAGTTT
yacH-F	GTCATACCATCCAATACAGT
yacH-R	GGAATATAGACCACATCAGG
narG-F	ATACGGTGAAATCCCTGAAA
narG-R	TTAGAACGCCAGCTGAACAG
actP-F	TTGTTACCACCAATCAGATG
actP-R	GGTTTATGGGCTACTTCTAT
yhbO-F	TGCCGTTTTAATCACTGATG
yhbO-R	TTTGCCTTTCACCGTTTTAC

Table S3. Primers in knockout experiments with the CRISPR/Cas system

Primers	Sequence(5'– 3')[a]
TargetF-R	ACTAGTATTATACCTAGGACTGAG
pTargetF-IR	ACTGCGGAGCCGTACAAATG
glgSN20	**GTATTGTCAACAGATGATGC**
glgSgF	AGTCCTAGGTATAATACTAGTGTATTGTCAACAGATGATGCGTTTTAGAGCTAGAAATAG
glgSDLF	CCGGTATAATGCACCGCAATAATCG
glgSDLR	ATCAGGCCCGTTATAAAGCACATCCTCCGGT
glgSDRF	ACCGGAGGATGTGCTTTATAACGGGCCTGAT
glgSDRR	AAGGTATGAGCGGAGCAGGTGCTA
yibTN20	**TTATCGGCCGAAGCAGGTAG**
yibTgF	AGTCCTAGGTATAATACTAGTTTATCGGCCGAAGCAGGTAGGTTTTAGAGCTAGAAATAG
yibTDLF	TAATTGCGCGCTGGAATGGCA
yibTDLR	AGTAAGCAACGTCTGCGATGATATCTCCGTAT
yibTDRF	ATACGGAGATATCATCGCAGACGTTGCTTACT
yibTDRR	CTTCTGGTGCTTCAGGTATTCCGC

[a]The black bold sequences indicated that the N20 sequences of target genes.

Table S4. Primers of overexpression experiment

Primers	Sequence(5'– 3')
ACS-F	CCGTTTTTTTGGGCTAGCGAATTCGAGCTCCAAGGAGAACAAAAGCATGAGC
ACS-R	ATCTTCTCTCATCCGCCAAAACAGCCAAGCTTTTACGATGGCATCGCGATAG
ACTP-F	TTTTTGGGCTAGCGAATTCGAGCTCATGAGGTACAAGCATCATGAAAAGAGTTCT
ACTP-R	CATCCGCCAAAACAGCCAAGCTTTTAATGCGCGCGGCCTTGCTCAA
ASTC-F	CCGTTTTTTTGGGCTAGCGAATTCGAGCTCTACTGTAGAGGTCGCTATGTCTCAG
ASTC-R	ATCTTCTCTCATCCGCCAAAACAGCCAAGCTTTCATGATGAACCTCGGCTAAC
ELAB-F	CCGTTTTTTTGGGCTAGCGAATTCGAGCTCCAAATGGAGAACGAGAATGTCTAAT
ELAB-R	ATCTTCTCTCATCCGCCAAAACAGCCAAGCTTTTAACGGCGTGCCAGCAACA
FADA-F	CCGTTTTTTTGGGCTAGCGAATTCGAGCTCGGCTTAAGGAGTCACAATGGAA
FADA-R	ATCTTCTCTCATCCGCCAAAACAGCCAAGCTTTTAAACCCGCTCAAACACCG
FADB-F	CCGTTTTTTTGGGCTAGCGAATTCGAGCTCATGCTTTACAAAGGCGACACC
FADB-R	CTTCTCTCATCCGCCAAAACAGCCAAGCTTTTAAGCCGTTTTCAGGTCGC
FADL-F	CCGTTTTTTTGGGCTAGCGAATTCGAGCTCCATTGAGGTTATGGTCATGAGC
FADL-R	ATCTTCTCTCATCCGCCAAAACAGCCAAGCTTTCAGAACGCGTAGTTAAAGTTAGTA
GADE-F	ACCCGTTTTTTTGGGCTAGCGAATTCGAGCTCGGCTAAGGAGCAAGTTATGATTTT
GADE-R	CTTCTCTCATCCGCCAAAACAGCCAAGCTTCTAAAAATAAGATGTGATACCCAGGG
HDEB-F	CCGTTTTTTTGGGCTAGCGAATTCGAGCTCTATTGAATGGGTTACAAAATGAA
HDEB-R	CTTCTCTCATCCGCCAAAACAGCCAAGCTTTTAATTCGGCAAGTCATTAGAT
SLP-F	CCGTTTTTTTGGGCTAGCGAATTCGAGCTCGTTGATAAGGATAGTAACATGAA
SLP-R	CTTCTCTCATCCGCCAAAACAGCCAAGCTTTTATTTGACCAGCTCAGGTGT
YHBO-F	ACCCGTTTTTTTGGGCTAGCGAATTCGAGCTCACAACGCGGAGGAAGCATGAGTAAGA
YHBO-R	CTTCTCTCATCCGCCAAAACAGCCAAGCTTTCAGGCACCGAGCAGGCGTAAC
YIAG-F	CCGTTTTTTTGGGCTAGCGAATTCGAGCTCATTTGAGGAGTTTTCAATGGAAT
YIAG-R	ATCTTCTCTCATCCGCCAAAACAGCCAAGCTTCTATTCCATCAACTGCTTACTTAATG
YJCH-F	ACCCGTTTTTTTGGGCTAGCGAATTCGAGCTCCTCTGGAGAATCTGTGATGAATG
YJCH-R	CTTCTCTCATCCGCCAAAACAGCCAAGCTTTCATGATGCTTGTACCTCATGC
GLGS-F	CCGTTTTTTTGGGCTAGCGAATTCGAGCTCACCGGAGGATGTGCTTATGGAT
GLGS-R	ATCTTCTCTCATCCGCCAAAACAGCCAAGCTTTCAGTGCTCTAACTCCAGCTCTCTTG
YIBT-F	CCGTTTTTTTGGGCTAGCGAATTCGAGCTCATACGGAGATATCATCATGGGCA

YIBT-R	ATCTTCTCTCATCCGCCAAAACAGCCAAGCTTTACTGCCCCTCTACCTGCTTC
pBADIup	CACACTTTGCTATGCCATAGCA
pBADIdown	ACCGCTTCTGCGTTCTGATT

Table S5. The genes were selected to delete or over express

Gene	Function	Up or down regulated	Deletion or overexpression
glgS	glycogen synthase surface composition regulator/ motility and biofilm regulator	down	D
yibT	uncharacterized protein<br>hypothetical protein	down	D
acs	acetyl-coenzyme A synthetase	up	O[*2]
actP	cation acetate symporter	up	D/O
astC	succinylornithine aminotransferase	up	O
elaB	DUF883 family protein, putative membrane-anchored ribosome-binding protein	up	O
fadA	acetyl-CoA C-acyltransferase FadA 3-ketoacyl-CoA thiolase (thiolase I)	up	O
fadB	fatty acid oxidation complex subunit alpha FadB	up	D/O
fadL	long-chain fatty acid transporter	up	O
gadE	transcriptional regulator GadE	up	O
hdeB	acid stress chaperone HdeB	up	O
slp	outer membrane protein slp	up	O
yhbO	stress-resistance protein	up	D/O
yiaG	transcriptional regulator	up	O
yjch	DUF485 family inner membrane protein	up	D/O

[*1] deletion, [*2] overexpression

Table. S6 Summary of available *E. coli* genes that involved in n-butanol tolerance.

Classification	Genes[a]	Reference
Oxidative stress related genes	*sodA, sodC* and ***yqhD***	(Rutherford et al., 2010; Si et al., 2016)
Cell envelope stress related genes	*rpoE, clpB* and *spy*	(Rutherford et al., 2010)
Chaperons	*htpG* and *dnaJ*	(Rutherford et al., 2010)
Stress repose genes (heat shock, organic solvent, acid, cold shock)	*cpxR, degP, rpoH, rpoE,* ***yibA***, ***metA***, ***ymcE,*** *yjcD, astE, ygiH rph, phoB/R* and *raiA*	(Rutherford et al., 2010; Reyes et al., 2011)
Respiratory related genes	*nuo operons* and *cyo operons*	(Rutherford et al., 2010)
Metabolite transport and biosynthesis genes	*hisJ, cysD, leuD, glnH, gadA, potF, oppABCDF, argO, emrA, argD, argR, dapD, lysC, leuA, leuB, kdpB, luxS, purP, manX, manY,* ***malE***, ***phnH***, ***metE*** **and *alsB***	(Rutherford et al., 2010)

Regulators	*rseA*, *crl*, *soxS*, *srmB*, *rpoD*, *rpoN*, *rplP*, *rplC*, *rpiB* , *rpsF,* ***cpxP*, *evnY*** and ***evgS***	(Rutherford et al., 2010)
Efflux pump system genes	*marA*, *mdtB*, ***focA*** and ***acrB***	(Rutherford et al., 2010; Reyes et al., 2011)
Membrane proteins related genes	*ompX*, *smpA*, *ompG*, ***yibT***, ***yghW***, ***Gcl***, ***glcF***, ***ybjC***, ***ompF***, ***yfdG*** and ***ompT***	(Rutherford et al., 2010; Reyes et al., 2011)
Iron metabolism related genes	*allB, metK, pdxA, araA, leuB, menD, pphA, pykF,* ***feoA* and *entC***	(Rutherford et al., 2010; Reyes et al., 2011)
Energy metabolism	***hyaF***	(Reyes et al., 2011)

[a]The black bold genes indicated that it has been confirmed to be related to butanol tolerance by overexpression or knockout experiment.

References

Datsenko, K.A., and Wanner, B.L. (2000). One-step inactivation of chromosomal genes in Escherichia coli K-12 using PCR products. *Proceedings of the National Academy of Sciences of the United States of America* 97(12)**,** 6640-6645.

Guzman, L.M., Belin, D., Carson, M.J., and Beckwith, J. (1995). Tight regulation, modulation, and high-level expression by vectors containing the arabinose PBAD promoter. *Journal of Bacteriology* 177(14)**,** 4121. doi: 10.1128/jb.177.14.4121-4130.1995.

He, X., Xue, T., Ma, Y., Zhang, J., Wang, Z., Hong, J., et al. (2019). Identification of functional butanol-tolerant genes from Escherichia coli mutants derived from error-prone PCR-based whole-genome shuffling. *Biotechnology for Biofuels* 12(1)**,** 73. doi: 10.1186/s13068-019-1405-z.

Jiang, Y., Chen, B., Duan, C.L., Sun, B.B., Yang, J.J., and Yang, S. (2015). Multigene Editing in the Escherichia coli Genome via the CRISPR-Cas9 System. *Applied and Environmental Microbiology* 81(7)**,** 2506-2514.

Reyes, L.H., Almario, M.P., and Kao, K.C. (2011). Genomic Library Screens for Genes Involved in n-Butanol Tolerance in Escherichia coli. *Plos One* 6(3).

Rutherford, B.J., Dahl, R.H., Price, R.E., Szmidt, H.L., Benke, P.I., Mukhopadhyay, A., et al. (2010). Functional genomic study of exogenous n-butanol stress in Escherichia coli. *Appl Environ Microbiol* 76(6)**,** 1935-1945. doi: 10.1128/AEM.02323-09.

Si, H.M., Zhang, F., Wu, A.N., Han, R.Z., Xu, G.C., and Ni, Y. (2016). DNA microarray of global transcription factor mutant reveals membrane-related proteins involved in n-butanol tolerance in Escherichia coli. *Biotechnology for Biofuels* 9.
